# Delayed Dental Development in Children With Non‐Syndromic Hypodontia: A Cross‐Sectional Study Using a Machine Learning Approach to Dental Age Estimation

**DOI:** 10.1111/ocr.70089

**Published:** 2025-12-29

**Authors:** Marine Crosnier, Pierre‐Hadrien Decaup, Frédéric Santos, Anaïs Cavare

**Affiliations:** ^1^ UFR des Sciences Odontologiques Univ. De Bordeaux Bordeaux France; ^2^ CNRS, MCC, PACEA, UMR 5199 Univ. De Bordeaux Pessac France; ^3^ Centre de Compétence des Maladies Rares Orales et Dentaires CCMR O‐Rares, C.H.U. de Bordeaux Bordeaux France

**Keywords:** dental age, dental developmental stage, hypodontia, machine learning, tooth agenesis, tooth agenesis pattern

## Abstract

**Objectives:**

To investigate the influence of non‐syndromic hypodontia on radiographic dental development and to estimate dental age in children with bilateral mandibular agenesis using a machine learning approach.

**Materials and Methods:**

This retrospective cross‐sectional study included 626 children aged 6–15 years (311 with dental agenesis, 315 matched controls). Dental age (DA) was assessed using the original Demirjian method. In cases with bilateral congenitally missing teeth in the mandible, DA was estimated using supervised machine learning models based on specific random forests, following a secondary‐diagnosis approach. Dental developmental delay was calculated as the difference between dental and chronological age (DA–CA) and compared across groups defined by agenesis status, severity and pattern. Multiple linear regression was applied to evaluate the effects of hypodontia, sex, chronological age and their interactions on DA–CA.

**Results:**

Eight random‐forest models were trained, achieving good age‐prediction accuracy (MAE = 0.08–0.28 years, R^2^ > 0.95). A 0.77‐year difference in dental development (95% CI 0.61–0.94) separated children with hypodontia from controls (*p* < 0.001). The regression model confirmed that agenesis status, sex and CA were significant predictors of DA–CA, with an interaction between sex and agenesis. Unilateral or bilateral agenesis of mandibular second premolars was associated with a dental developmental delay compared with controls (*p*_*adj*_ < 0.05).

**Conclusion:**

Our results were consistent with broader evidence linking hypodontia to altered developmental timing. Machine learning imputation offers a robust approach for missing teeth and can be implemented for age estimation in larger cohorts for orthodontic or forensic purposes.

## 1 Introduction

Tooth agenesis is defined as the congenital absence of one or more teeth, in the primary or permanent dentition and can occur either in isolation or as part of a broader genetic syndrome. The prevalence of congenitally missing teeth (CMT), excluding third molars, ranges from 5.7% to 7.2% in the permanent dentition [1], with significant variations depending on ethnicity and sex [1, 2, 3].

Dental development is a multidimensional process influenced by genetic, epigenetic and environmental interactions under precise spatiotemporal control [4]. Polymorphisms in genes critical to odontogenesis may disrupt developmental signalling pathways or exert pleiotropic effects, resulting in the co‐occurrence of anomalies affecting tooth number, size and shape [5]. Additional anomalies, including infraocclusion of primary molars, tooth transpositions, palatal displacement of canines and delayed eruption, have also been integrated into a broader concept known as the Dental Anomaly Pattern [6]. Garn et al. proposed that third molar agenesis represents the most extreme expression of factors delaying tooth formation, suggesting that disturbances during odontogenesis increase the likelihood that later‐developing teeth fail to form entirely [7]. Indeed, understanding these co‐variations may not only help identify common developmental aetiologies but also reinforce the evolutionary theory of developmental disruptions [8]. From a clinical perspective, strengthening the available evidence could improve orthodontic treatment planning, as the choice and timing of orthodontic appliances are influenced by dental and skeletal developmental stages.

Several clinical studies have reported a significant delay in dental development in individuals with non‐syndromic hypodontia (regardless of the type of CMT) compared to control groups. The observed delays (calculated as dental age minus chronological age) range from 0.3 to 1.51 years depending on the study [9, 10, 11, 12]. Other authors have also reported delays in dental maturation stages of the remaining teeth in cases of hypodontia [13, 14, 15, 16]. On the other hand, conflicting results have been reported, with some studies reporting no significant differences [17, 18, 19, 20] or identifying delays only in cases of oligodontia [21]. This inconsistency may arise from variations in the geographical background of the studies or the methods employed to assess dental age. Furthermore, evident disparities exist in the approaches used to quantify delays in dental development [22, 23, 24, 25].

One major limitation in previous studies is the lack of an accurate method for estimating dental age in patients with congenitally missing teeth, as maturity scales do not account for missing data and are not applicable in cases of bilateral agenesis. Several approaches have been explored, such as excluding patients with bilateral agenesis [12], applying modified versions of the Demirjian method based on fewer teeth [10] or substituting a maxillary tooth when feasible [23]. The Haavikko method is often preferred, as it enables the calculation of mean dental age from the teeth present, though this approach raises concerns regarding comparability with control groups [9, 11, 26]. To the best of our knowledge, no study has yet applied a regression‐based machine learning approach specifically to hypodontia cases, despite promising results reported for dental age estimation in the general population [27].

The present study aimed to compare dental development relative to chronological age between healthy children and those affected by non‐syndromic hypodontia in a French orthodontic population and to assess the potential influence of biological sex, chronological age and patterns of tooth agenesis on this delay. The null hypothesis (H0) was that there is no difference in dental developmental delay (defined as the difference between dental age and chronological age) between children with and without non‐syndromic hypodontia. Machine learning models were also developed to predict dental age in individuals with bilateral mandibular CMT.

## 2 Materials and Methods

This study follows the recommendations provided by the STROBE (Strengthening the Reporting of Observational Studies in Epidemiology) guidelines for presenting observational studies [28].

### 2.1 Study Design and Participants

In this retrospective cross‐sectional study, the sample size was estimated for the primary outcome variable, defined as the difference between dental age and chronological age, using R statistical software version 4.5.1 (R Core Team, 2024, Vienna, Austria) along with the *pwr* package. A minimum of 124 children was required to detect a significant difference using a two‐sample t‐test, with a type I error (α) of 0.05, a statistical power (1‐β) of 90% and an effect size (Cohen's *d*) of 0.413, derived from the findings of León‐Rubio et al. [12].

The hypodontia group consisted of a previously described sample of 311 children, aged between 6 and 15 years, with agenesis of one or more permanent teeth, excluding third molars [29]. For the present analysis, the first panoramic radiograph taken before orthodontic treatment was assessed. Additionally, radiographs taken at an age older than 9 years were examined to confirm dental agenesis. This sample was matched with a control group of non‐affected children of the same sex, age and geographic origin. Exclusion criteria were patients with syndromes or diseases affecting craniofacial morphology, incomplete medical records, or poor‐quality panoramic radiographs that compromised the identification of dental agenesis or the assessment of tooth maturation stages.

All panoramic radiographs were obtained from orthodontic patient records collected over a 16‐year period (2006–2022) from a private practice in the South West of France. Approval was obtained from the Research Ethics Committee of the University Hospital of Bordeaux (CER‐BDX 2023‐133). Written consent from the legally responsible carer of the child was obtained and data were anonymised twice. All identifying information was removed at data extraction and a unique anonymised participant code was subsequently assigned to each case, preventing any re‐identification during the analysis.

### 2.2 Data Collection

The evaluation of digitised patient panoramic radiographs was conducted by one trained examiner (Author 1), blinded for the chronological age, between May 2024 and September 2024. Each subject contributed only once to the dental development analysis, eliminating repeated observations.

Tooth agenesis had already been assessed in the aforementioned publication but was re‐evaluated for each patient to ensure accuracy [29]. The number of CMT per individual was recorded and the severity of agenesis was categorised as mild (1–2 CMT), moderate (3–5 CMT), or oligodontia (six or more missing teeth). The configurations of tooth agenesis among the affected patients were represented using the TAC tool, a binary code developed by van Wijk and Tan [30]. Each CMT was assigned a ‘tooth value’ and each dental quadrant was analysed separately. The sum of these values within each quadrant generated a specific value that characterised a unique tooth agenesis profile.

### 2.3 Dental Development Assessment

Dental age (DA) was estimated using the original Demirjian method [31]. The developmental stages of the seven permanent teeth in the lower left quadrant of the mandible, from the central incisor to the second molar, were scored according to the eight radiographic stages (A to H). When a tooth was congenitally missing, the lower right quadrant was analysed instead. Each alphabetical stage was converted into a numerical dental maturity score using standard sex‐specific tables. The scores for each individual were then summed to obtain a global maturity score. Finally, this global maturity score was converted into DA, expressed in years, using sex‐specific reference tables.

When a tooth was bilaterally missing, the stage was recorded as missing and DA was predicted using a supervised machine learning regression algorithm. This model relied on developmental stages and DA calculated from the remaining sample, following the principle of secondary diagnosis [32]. All analyses were performed using R 4.5.1. Annotated code is provided in a step‐by‐step workflow in Supporting Information, along with the complete machine learning results presented in Figures S1–S6 and Tables S1–S3.

**FIGURE 1:**
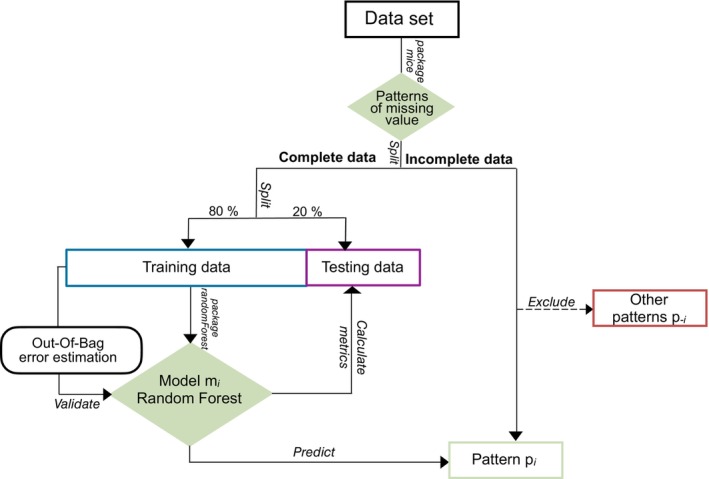
Overview of the modelling process for dental age prediction in subjects with bilateral mandibular agenesis using Random Forest.

The modelling process adopted an iterative approach for each specific pattern of missing stage ratings. A dedicated pipeline was implemented for each missing pattern to train a Random Forest (RF) model for DA estimation based on the developmental stages of the remaining lower left permanent teeth and sex (Figure 1). For each pattern, individuals with fully staged teeth and a calculated DA (from either the control or agenesis group) formed the dataset used for model training (80%) and testing (20%). Each RF model was trained to predict DA (target variable) based on the developmental stages of the remaining teeth and sex (predictor variables).

Each pipeline used the random forest's native out‐of‐bag (OOB) error to prevent overfitting during hyperparameter optimisation. Model performance was assessed using standard metrics on the test set, including the coefficient of determination (R^2^), mean error (ME), root mean square error (RMSE), mean square error (MSE) and mean absolute error (MAE). Additionally, Bland–Altman plots were used to graphically evaluate the agreement between the reference DA and the predicted values.

Once validated, the model was applied to estimate DA for individuals corresponding to the given pattern of missing ratings. Individuals not matching this pattern were excluded and assigned to their respective RF model.

The chronological age (CA) of each subject was calculated as the number of years between the date of the panoramic radiograph and the date of birth and was recorded to one decimal place. Delay in dental development was obtained by the difference between dental (DA) and chronological age (CA) or DA–CA.

### 2.4 Statistical Analysis

Statistical analysis was performed using R software along with the packages *prettyR*, *car* et *FSA*. Descriptive statistics were calculated for the variables of interest. The choice between parametric and non‐parametric tests was based on the results of Shapiro–Wilk and Levene's tests for normality and homogeneity of variances.

The Student's *t*‐test was used to compare the mean differences in DA, CA and dental delay (DA–CA) between the control group and the hypodontia group. Additionally, individuals were categorised as presenting marked dental developmental delay using a −2 SD cut‐off derived from the control group distribution of DA–CA. The distribution of delayed individuals was then compared between the two groups using a chi‐square test with Yates' continuity correction.

A multiple linear regression model was used to evaluate the effects of dental agenesis (presence or absence), sex and CA on DA–CA. A Type II ANOVA was then applied to this model to assess the global contribution of each factor and their interactions. Model validity was verified through residual‐based diagnostics, including homoscedasticity and linearity assessments via residuals versus fitted values plots and normality testing using the Shapiro–Wilk test. Scatter and boxplots were used to visually explore the relationship between DA–CA and chronological age, or agenesis severity and sex. Additionally, a Tukey HSD test was used for multiple comparisons between severity levels following a one‐way ANOVA on DA–CA.

To assess whether the dental agenesis profile (represented by the TAC value) influenced dental delay, a Kruskal–Wallis test was conducted on the entire sample, followed by Dunn's post hoc comparisons when appropriate.

The level of significance was 5% (*p* = 0.05). All *p*‐values were adjusted for multiple comparisons using the Bonferroni method.

### 2.5 Reproducibility

An intra‐observer reproducibility test for the use of tooth development stage scales (A to H) was conducted on 50 panoramic radiographs, randomly selected using the sample function in R and analysed by the same examiner after a two‐week wash‐out period. Cohen's Kappa coefficient was calculated using the R statistical software and the irr package to assess the agreement between two evaluations performed by the same examiner for each of the seven left mandibular teeth scored. The result of the intra‐observer test ranged between 0.887 and 1, indicating a perfect level of agreement (Table S4).

## 3 Results

The entire dataset included 626 subjects (360 females, 266 males) with a mean age of 11.72 ± 1.76 years. Among them, 311 were in the agenesis group (178 females, 133 males) and 315 in the control group (182 females, 133 males), with mean ages of 11.57 ± 1.86 and 11.87 ± 1.64 years, respectively. No significant differences were found between groups regarding chronological age (*p* = 0.06, W‐test) or sex distribution (*p* = 0.90, χ^2^ test).

**FIGURE 2:**
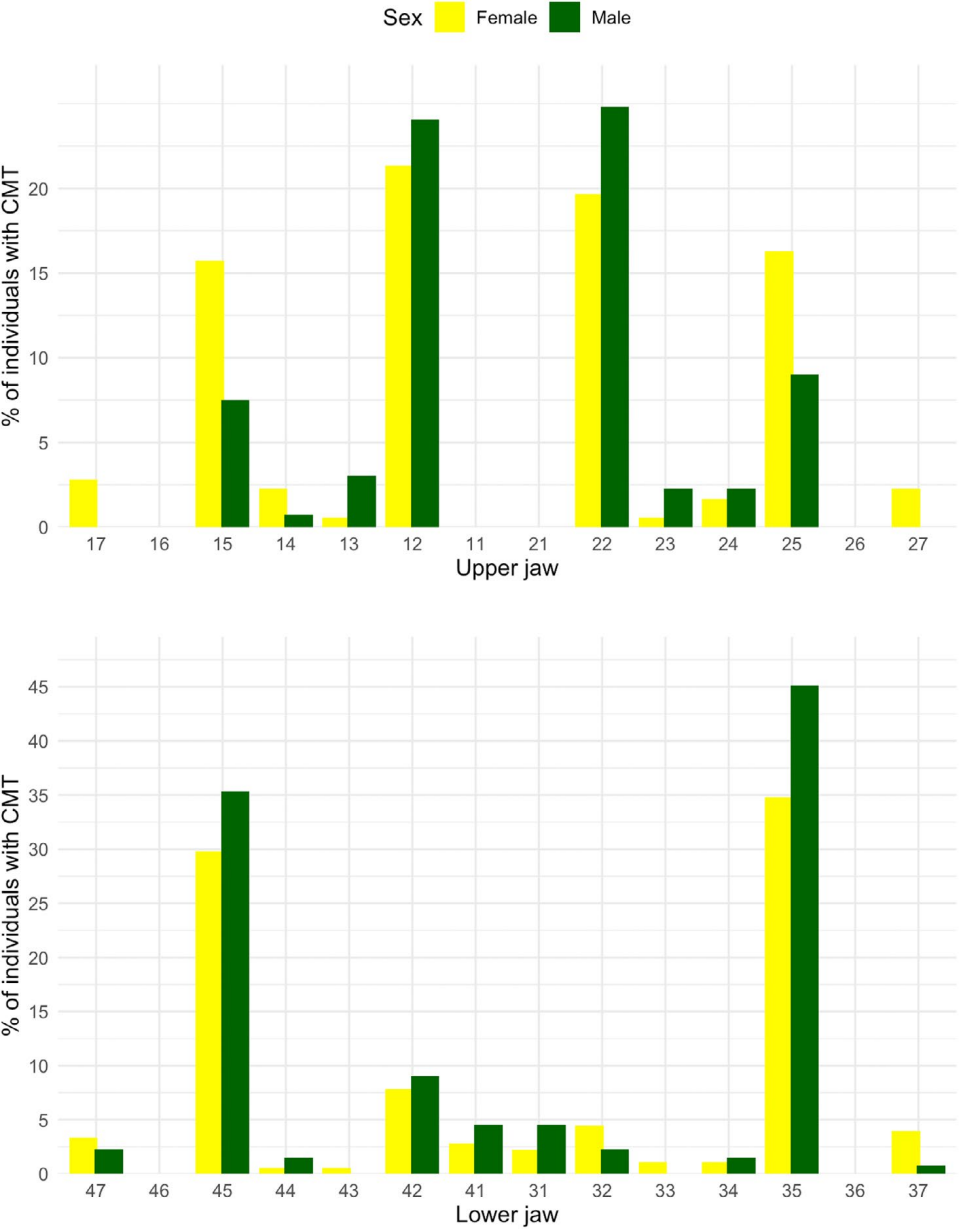
Sex‐specific distribution of each single tooth agenesis. Percentages are calculated relative to the number of affected individuals per sex.

A total of 552 teeth were congenitally missing, with a mean of 1.78 ± 1.20 missing teeth per affected subject (range: 1–8). In the agenesis group, most individuals had one (51.4%) or two (34.7%) missing teeth, whereas moderate (11.3%) and oligodontia (2.6%) cases were less frequent. The detailed distribution of the number of missing teeth per subject is provided in Supplementary Table S5. Mandibular second premolars and maxillary lateral incisors were the most frequently missing teeth, with minor sex‐related differences (Figure 2).

The supervised machine learning approach for dental age estimation was applied to predict DA in 78 individuals with at least one bilaterally missing developmental stage. Eight different RF models were developed to account for the various patterns of missing stage ratings identified in the sample (Figure S1). These models were trained on a reference dataset of 548 complete individuals, split into a training set (*n* = 438) and a testing set (*n* = 110) for internal validation. All RF models demonstrated high reliability, with R^2^ values exceeding 95% and mean absolute errors ranging from 0.08 to 0.28 years (Tables S2, S3). The Bland–Altman plots suggested minimal bias across all models (Figures S5, S6), with differences between predicted and actual dental ages symmetrically distributed around zero and mostly confined within the 95% limits of agreement (±1.96 SD). No sex‐ or age‐related trends in prediction error were observed.

The difference between dental and chronological age (DA–CA) indicated delayed dental development in children with dental agenesis (−0.23 ± 1.02 years), whereas unaffected subjects showed a relative advancement (+0.54 ± 1.07 years). The comparison of DA–CA between groups was statistically significant (Table 1, *p* < 2e–16), resulting in rejection of the null hypothesis. This finding corresponded to an intergroup discrepancy in DA–CA between affected and control subjects estimated at 0.77 years (95% CI = 0.61–0.94). Similar trends were observed in the subgroup analysis by sex, with a larger difference between affected and control groups in females (0.94 years; 95% CI = 0.73–1.15) than in males (0.55 years; 95% CI = 0.28–0.81). Using a−2 SD cut‐off (DA–CA < −1.61 years), 7.7% of hypodontia patients and 1.6% of controls were classified as presenting a marked dental developmental delay, with a significant between‐group difference (χ^2^, *p* < 0.01).

**TABLE 1:** Chronological and dental ages and their differences (DA–CA) in individuals with and without dental agenesis.

								Differences in DA–CA between control and agenesis groups		
	CA		DA		CA vs DA	DA–CA		*p*	ΔDA–CA	
	Mean (years)	SD	Mean (years)	SD	*p*	Mean (years)	SD		Mean (years)	CI 95%
Total										
Control group	11.87	1.64	12.41	1.95	***< 2e‐16***	0.54	1.07	***< 2e‐16***	0.77	[0.61; 0.94]
Agenesis group	11.57	1.86	11.34	1.96	***5.9e‐5***	−0.23	1.02			
Females										
Control group	11.90	1.57	12.60	1.83	***< 2e‐16***	0.70	1.00	***< 2e‐16***	0.94	[0.73; 1.15]
Agenesis group	11.51	1.92	11.27	1.99	***0.002***	−0.24	1.01			
Males										
Control group	11.82	1.73	12.14	2.09	***0.002***	0.32	1.14	***5.9e‐5***	0.55	[0.28; 0.81]
Agenesis group	11.66	1.77	11.43	1.92	***0.01***	−0.23	1.05			

*Note:* Significant differences (*p* < 0.05) are indicated in bold italics (Student *t*‐test).

Abbreviations: CA, Chronological age; CI Confidence Interval; DA: Dental age; SD, Standard Deviation.

The multiple linear regression model showed a significant association between DA–CA and the presence of agenesis, defined as a dichotomous variable, as well as with sex (Table 2). A significant interaction between sex and agenesis suggested that the effect of agenesis on DA–CA differed between males and females. Chronological age was also a significant predictor, although its interactions with other variables were not. Visual inspection of residuals versus fitted values did not reveal any major violations of linearity or homoscedasticity and the distribution of residuals was normal (Shapiro–Wilk test: *p* = 0.087). These findings support the validity of the model.

**TABLE 2:** Type II ANOVA table from the multiple linear regression model predicting dental delay (DA–CA) from agenesis status, sex, chronological age and their interactions.

	Sum of squares	DF	*F*	*p*
Predictors				
Agenesis	97.54	1	90.598	***< 2e‐16***
Sex	5.32	1	4.937	***0.027***
CA	5.75	1	5.337	***0.021***
Agenesis: Sex	6.46	1	5.997	***0.015***
Agenesis: CA	3.36	1	3.123	0,078
Sex: CA	0.30	1	0.279	0,598
Agenesis: Sex: CA	0.06	1	0.053	0,818
Residuals	665.35	618		

*Note:* Significant differences (*p* < 0.05) are indicated in bold italics.

Abbreviations: CA, Chronological age; DF, degrees of freedom.

**FIGURE 3:**
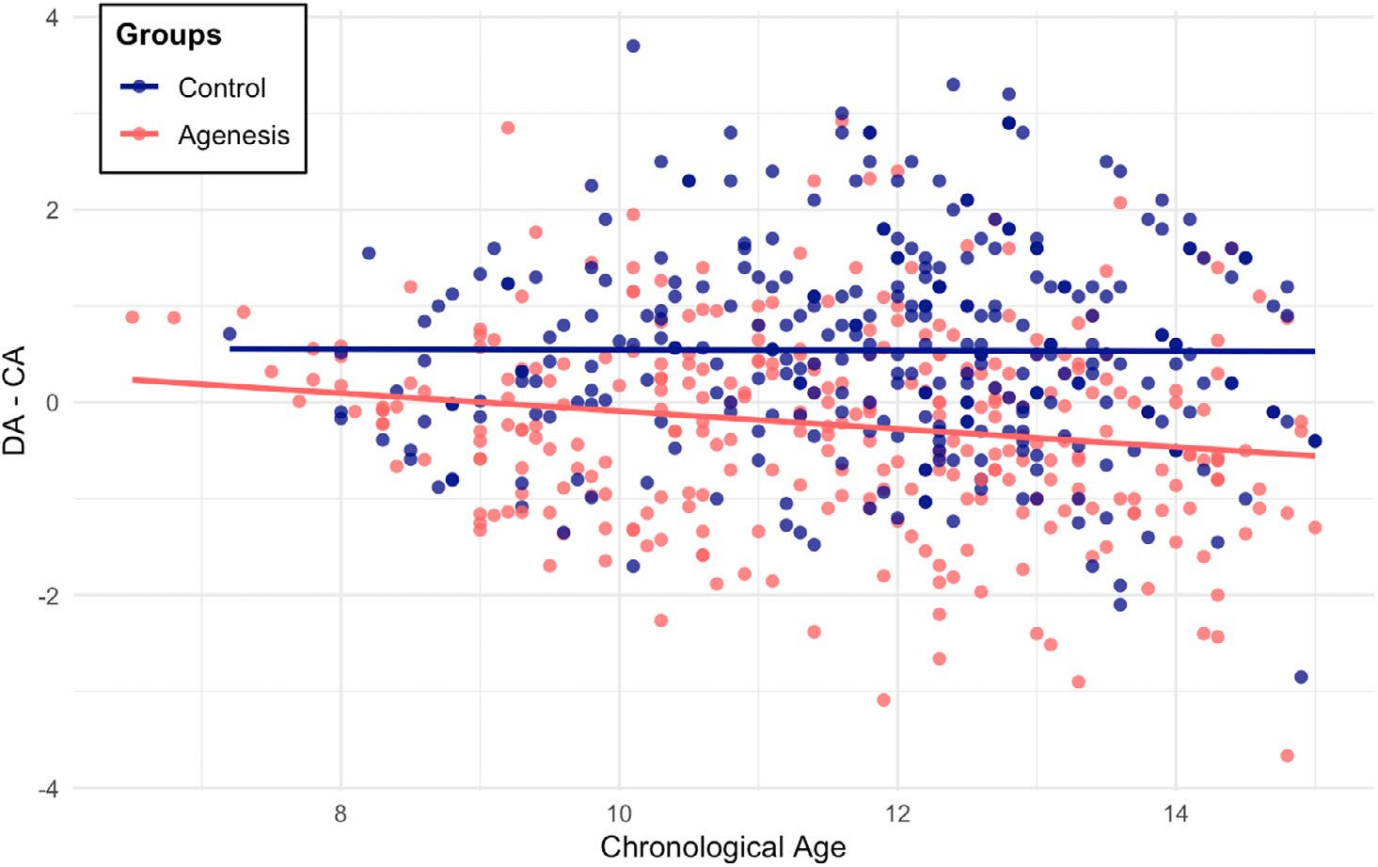
Scatterplot of the differences between dental age and chronological age versus. Chronological age for control and agenesis groups. Separate regression lines were used for each group, based on ANCOVA analysis showing that including agenesis status significantly improved the model fit (*p* < 0.001).

**FIGURE 4:**
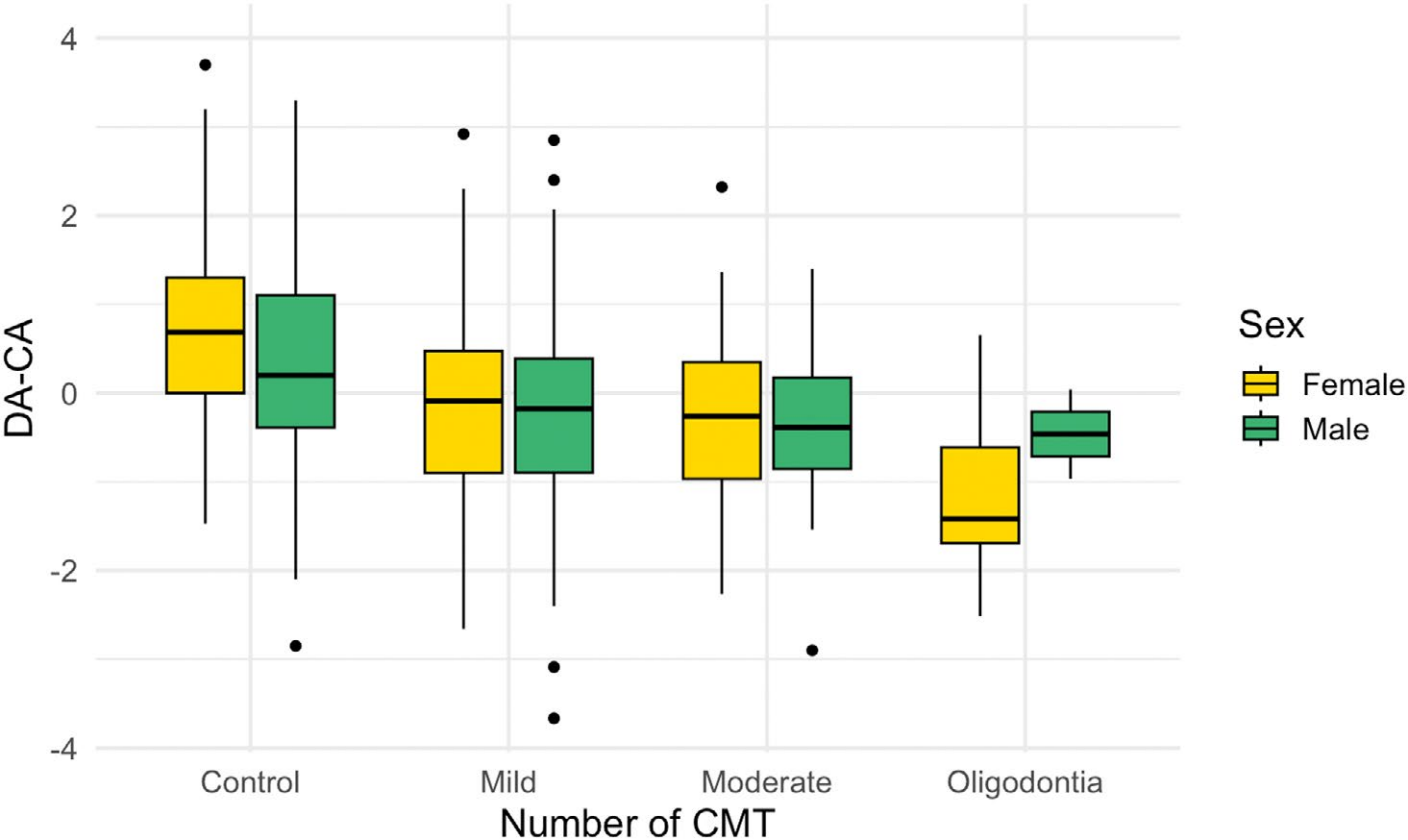
Boxplot of the differences between dental age and chronological age (DA–CA) according to the severity of dental agenesis, by sex. The Tukey HSD test revealed significant differences only between each agenesis severity group and the control group, not between severity levels themselves.

This age‐related increase in dental delay is illustrated in Figure 3, particularly among the affected group, despite the corresponding interaction not reaching statistical significance in the aforementioned model. DA–CA exhibited a progressive increase with the severity of agenesis, based on the number of CMT (Figure 4). Sex‐related differences in DA–CA appeared to decrease in cases of mild and moderate hypodontia.

A total of 63 distinct configurations of dental agenesis, based on TAC values, were identified in the sample (Table S6). A significant difference in DA–CA was found across these patterns (Kruskal–Wallis test, *p* = 4.8e–07). Post hoc pairwise comparisons indicated that individuals with unilateral or bilateral agenesis of the mandibular second premolars exhibited significantly greater delays in dental development, but only in comparison with controls (Dunn's test: 35: *p*_*adj*_ = 0.009; 45: *p*_*adj*_ = 0.011; 35 and 45: *p*_*adj*_ = 0.002).

## 4 Discussion

This study has several strengths supporting the reliability of its findings. The age range (6–15 years) is consistent with most previous studies [10, 11, 12, 18, 19, 25] and the sample size (*n* = 626) exceeds that of earlier research on hypodontia and dental delay [9, 10, 11, 12, 17, 18, 26, 33], as well as the number required based on prior effect‐size estimates.

The machine learning approach enabled inclusion of 78 individuals (25% of the hypodontia group) who would otherwise have been excluded, thereby maintaining statistical power and ensuring comprehensive representation of agenesis patterns. Among studies estimating dental age without excluding participants, rigorous validation is often lacking, leaving the precision of these methods uncertain. Nyström et al. [34] proposed conventional regression equations to impute the stage of a missing mandibular tooth using the six remaining teeth, based on a Finnish cohort. However, reported accuracy was moderate (68.6%–83.3%) depending on the tooth missing. Dhamo et al. [23] later used these equations in a Dutch cohort, but without evaluating predictive accuracy beyond limited comparison with maxillary substitution, leaving their performance unverified.

Artificial intelligence methods for age estimation are typically divided into two approaches: machine learning using human staging inputs (such as Demirjian scores) and deep learning, which extracts features directly from radiographs. A recent meta‐analysis [27] reported that AI models (particularly convolutional neural networks) achieve MAE between 0.4 and 0.9 years, although outlier studies contributed to heterogeneity, with errors up to 12 years depending on age group and dataset. Among machine learning models, random forest is a tree‐based ensemble method that uses bootstrap aggregation and random feature selection to produce robust, accurate predictions while reducing overfitting [35]. In our study, random forest models demonstrated high predictive performance, with MAE ranging from 0.08 to 0.28 years. In a recent comparative study using Demirjian inputs on a southwestern French cohort demographically similar to ours [36], random forest achieved the second‐best performance (MAE = 0.731 ± 0.025 years). However, the target variable was chronological age and bilateral agenesis cases were excluded. To the best of our knowledge, the i‐Dent project [37] is the only study to address age estimation in hypodontia using artificial intelligence. By combining CNN architectures to analyse tooth emergence patterns, the model achieved an accuracy of approximately 72%, but was trained on a highly specific population (syndromic or severe oligodontia cases). It could be argued that our sample size was limited and no external validation set was used, potentially reducing generalisability. To mitigate the risk of overfitting, we implemented internal validation strategies, including the use of out‐of‐bag (OOB) error during model training and an independent testing set that was never used during model fitting. Nevertheless, this approach should be extended to larger and more diverse cohorts to allow proper external validation. Moreover, our goal was to address missing data using reference individuals from a biologically homogeneous cohort, following the example of' ‘secondary sex diagnosis’ as used in biological anthropology [32]. This assumption of biological homogeneity represents another limitation, as it is inferred solely from geographical origin. Nevertheless, a recent genome‐wide study identified a southwestern genetic cluster corresponding to the same region as our study, which should be considered when interpreting our results [38]. By providing the full analytical pipeline as reproducible open‐access code, we aim to contribute to ongoing research on machine learning‐based age prediction and to enable independent validation of our results.

A significant dental developmental delay (mean difference in DA–CA) of 0.77 years (95% CI = 0.61–0.94) was observed in children with non‐syndromic hypodontia compared with controls, in line with earlier studies. Dhamo et al. [23] partially summarised these findings and proposed a mean deviation of 1.17 (95% CI = 1.07–1.27). Comparisons across studies remain difficult due to differences in dental staging methods, analytical approaches and population characteristics. However, because each study compares affected and control groups using the same staging standard, internal validity is maintained. Among studies that employed the Demirjian method, Ruiz‐Mealin et al. [26] reported significant delays of 0.84 years in boys and 0.87 years in girls. Kan et al. [33] similarly found delays of 0.9 years in boys and 1.1 years in girls. In contrast, Tunç et al. [10] observed a smaller but still statistically significant delay of 0.3 years in both sexes. León‐Rubio et al. [12] reported a significant delay of 0.50 years only in girls. Studies relying on Haavikko's 12‐stage method tend to report larger delays, ranging from 1.53 to 1.8 years in boys and 1.49 to 2.0 years in girls [9, 22]. Despite methodological variations, our findings reinforce the broader evidence of a strong association between hypodontia and delayed dental development across diverse populations. Furthermore, we observed that the magnitude of delay increased with the severity of dental agenesis (Figure 4), as also reported in prior research [9, 11, 19, 26], despite some conflicting evidence in the literature [10, 22]. Ruiz‐Mealin et al. [26] quantified this association, estimating an additional delay of 0.13 years for each missing tooth. Interpretation of findings in individuals with oligodontia should be made with caution, as they are underrepresented in most cohorts due to their rarity and the difficulty of estimating dental age when multiple teeth are congenitally absent. As no more than two mandibular stage ratings were missing per individual in our dataset, dental age was not estimated using random forest models in cases with more extensive agenesis, for which a lower prediction accuracy would be expected.

The present study simultaneously evaluated hypodontia, sex and chronological age as predictors of dental developmental delay (DA–CA) using multiple linear regression. To our knowledge, no previous research has assessed both main and interaction effects of these variables, offering a more integrated understanding of their respective influences. Sex was a significant predictor of dental development. In the control group, girls exhibited a greater positive DA–CA difference than boys, suggesting more advanced dental development. This sex‐based variation, reported in the literature [34], may reflect differential developmental timing or a slight overestimation of dental age in females. Beyond this effect, we also identified a significant interaction between sex and hypodontia, indicating that the magnitude of dental developmental delay associated with hypodontia was greater in girls than in boys. Although modelling differences limit comparisons, Tunç et al. found a significant delay only in boys [10], whereas no sex‐related differences have been reported by other authors among individuals with hypodontia [19, 20, 22, 26]. Kan et al. [33] hypothesised that the greater delay observed in girls during late childhood might reflect hormonal influences linked to pubertal timing. However, our data did not support this explanation: while chronological age significantly influenced DA–CA, no interaction was found between age and sex or between age and hypodontia and the three‐way interaction was likewise non‐significant. These findings challenge the notion that puberty‐related hormonal changes disproportionately affect dental maturation and in particular in affected patients. The significant sex and dental agenesis interaction raises the question of biological mechanisms beyond puberty. While hormonal modulation remains a plausible explanation, other pathways should be considered such as sex‐linked genetic regulation of odontogenesis. Interestingly, our previous findings revealed no sexual dimorphism in hypodontia prevalence or pattern distribution in the population‐based sample [29]. This highlights the complex nature of hypodontia, a multidimensional developmental condition whose variable phenotypic expression extends beyond tooth absence and may include sex‐related differences.

Concerning the observed effect of chronological age on DA–CA, one possible explanation, as suggested by Maber et al. [39], is that older children may have fewer teeth eligible for staging, as apex closure limits the accuracy of dental development assessment. This age‐related limitation may accentuate the observed delay. Our variable importance analysis (Figure S4) indirectly supported this observation, showing that the first premolar was the most informative feature for age prediction across all random forest models when present, followed by the second molar. In contrast, sex contributed minimally to prediction accuracy, highlighting the predominant role of dental maturation patterns.

Additionally, Ruiz‐Mealin et al. [26] reported a significant association between chronological age and dental developmental delay in individuals with hypodontia, with the delay increasing by 0.48 years for each additional year of age, while no change in delay was observed in the control group. Although a similar trend can be observed upon visual inspection of Figure 3, our data did not reveal a statistically significant interaction between chronological age and hypodontia, as previously reported. This discrepancy may reflect stricter confounder control in our models or the slightly smaller number of individuals older than 14 years, potentially reducing statistical power in this subgroup.

In‐depth analysis of agenesis profiles highlighted that individuals missing mandibular second premolars were more strongly associated with delayed dental development compared to controls. While a statistical artefact could be considered, this explanation is unlikely: in our sample, bilateral agenesis of the maxillary lateral incisors was more frequent than that of the right mandibular second premolar, but it was not significantly associated with delay. This finding is consistent with broader evolutionary and developmental theories [8]. According to Butler's field theory, each class of teeth develops along a gradient, with the most distal tooth in each class being both more variable and more susceptible to developmental disturbances. Although out of the scope of our analysis, previous studies have shown that the absence of second premolars may be associated with delayed maturation of neighbouring teeth, with variation depending on their mesial or distal position [9, 15, 17]. Garn et al. [7] described a mesial‐to‐distal gradient in both tooth size and formation timing and interpreted such patterns as indicative of a global delay in somatic maturation, in which dental development may serve as a sensitive marker of environmental stress. Bermúdez de Castro [40] also suggested that tooth agenesis could reflect broader patterns of heterochronic change in human dentition, as part of a phylogenetic trend associated with life history changes in *Homo sapiens* . In addition, this association may reflect pleiotropic effects of key developmental genes, including *PAX9*, *MSX1* and *WNT10A*, which influence both dental agenesis and the timing of tooth formation [23].

The present study has some limitations and must be interpreted taking into account the biases due to its retrospective nature: radiographic parameters could not be fully standardised and panoramic imaging, although ethically appropriate for paediatric patients, introduces two‐dimensional distortion [41]. The examiner could not be blinded to group allocation, but excellent intra‐observer agreement mitigates the risk of scoring bias. Because recruitment was limited to a single centre, the sample comprised only French Caucasian children, which restricts the generalisability of the findings. Potential environmental modifiers were not recorded and therefore could not be incorporated into the models, limiting their explanatory scope. Among these factors, nutritional status, socioeconomic background and ethnic variation have been described as influencing dental maturation and may have contributed to the observed delay [42]. Finally, the cross‐sectional design does not permit causal inference.

## 5 Conclusion

Non‑syndromic hypodontia is associated with a clinically relevant delay in dental development that intensifies with age and severity and is modulated by sex. Accordingly, clinicians should be aware that the timing of orthodontic treatment in hypodontia patients may need to be individualised. The machine learning approach presented here offers a robust solution for age estimation when conventional scoring is challenged by missing data, with promising applications in orthodontic research, biological anthropology and forensic science.

## Author Contributions

Conceptualisation: Pierre‐Hadrien Decaup, Anaïs Cavare; methodology: Pierre‐Hadrien Decaup, Frédéric Santos, Anaïs Cavare; validation: Pierre‐Hadrien Decaup, Frédéric Santos, Anaïs Cavare; formal analysis: Marine Crosnier, Anaïs Cavare; data curation: Anaïs Cavare; writing, original draft preparation: Marine Crosnier, Pierre‐Hadrien Decaup, Frédéric Santos, Anaïs Cavare; writing, review and editing: Marine Crosnier, Pierre‐Hadrien Decaup, Frédéric Santos, Anaïs Cavare; Supervision: Pierre‐Hadrien Decaup, Anaïs Cavare.

All authors read and approved the manuscript before submission.

## Funding

The authors have nothing to report.

## Ethics Statement

Ethical approval was waived by the Research Ethics Committee of the University Hospital of Bordeaux (CER‐BDX 2023–133) in view of the retrospective nature of the study and all the procedures being performed were part of the routine care.

## Consent

Informed consent was obtained from the child's legally responsible carer and from all individual participants included in the study.

## Conflicts of Interest

The authors declare no conflicts of interest.

## Supporting information


**Figure S1:** Missing data patterns for the whole dataset
**Figure S2:** Missing data patterns for boys only
**Figure S3:** Missing data patterns for girls only
**Figure S4:** Variable importance for all eight random forest models, computed as the increase
**Table S1:** Results of dental age estimation by random forests for each individual with at least
**Table S1:** Results of dental age estimation by random forests for each individual with at least
**Table S1:** Results of dental age estimation by random forests for each individual with at least
**Table S2:** Summarises the results obtained with the training
**Table S2:** Performance metrics of all eight random forest models based on out‐of‐bag results
**Table S3:** Performance metrics of all eight random forest models for the testing sample
**Figure S5:** Bland–Altman plots for all eight random forest models in the training sample
**Figure S6:** Bland–Altman plots for all eight random forest models in the training sample
**Table S4:** Cohen's kappa coefficients for each mandibular tooth (I1, I2, C, PM1, PM2, M1, M2) and overall inter‐rater agreement (mean ± SD)
**Table S5:** Distribution of the number of congenitally missing teeth (CMT) per affected subject
**Table S6:** Distribution of tooth agenesis patterns by frequency

## Data Availability

The data that supports the findings of this study are available in the supporting information of this article.
