# Supplementary material for: Delayed Dental Development in Children With Non‐Syndromic Hypodontia: A Cross‐Sectional Study Using a Machine Learning Approach to Dental Age Estimation
Source: Orthod Craniofac Res. 2025 Dec 29;29(2):347–56. doi: 10.1111/ocr.70089 (PMC12972234; doi:10.1111/ocr.70089)
Supplement: Supplementary file 1 — Figure S1: Missing data patterns for the whole dataset Figure S2: Missing data patterns for boys only Figure S3: Missing data patterns for girls only Figure S4: Variable importance for all eight random forest models, computed as the increase Table S1: Results of dental age estimation by random forests for each individual with at least Table S1: Results of dental age estimation by random forests for each individual with at least Table S1: Results of dental age estimation by random forests for each individual with at least Table S2: Summarises the results obtained with the training Table S2: Performance metrics of all eight random forest models based on out‐of‐bag results Table S3: Performance metrics of all eight random forest models for the testing sample Figure S5: Bland–Altman plots for all eight random forest models in the training sample Figure S6: Bland–Altman plots for all eight random forest models in the training sample Table S4: Cohen's kappa coefficients for each mandibular tooth (I1, I2, C, PM1, PM2, M1, M2) and overall inter‐rater agreement (mean ± SD) Table S5: Distribution of the number of congenitally missing teeth (CMT) per affected subject Table S6: Distribution of tooth agenesis patterns by frequency [file OCR-29-347-s001.pdf]

Delayed Dental Development in Children with Non-Syndromic Hypodontia: A Cross–Sectional Study Using a Machine-Learning Approach to Dental Age Estimation

Supporting Information: R code for statistical analyses

[redacted] [redacted] [redacted]
[redacted]

Table of contents

1 Configuration and packages **1**

2 Load and refactor data **2**
2.1 Individual data 2
2.2 Tables of conversion of dental stages by sex 3

3 Compute the dental age in the dataset **5**
3.1 Convert stages into numeric values 5
3.2 Computation of dental age 7

4 Split the data according to sex **8**

5 Inspecting missing data patterns **8**

6 Dental age estimation using random forests **10**
6.1 Pipeline design 10
6.2 Train random forests models 11
6.3 Variable importance in random forests models 12
6.4 Results of dental age estimation 13

7 Model diagnostics **15**
7.1 Metrics 15
7.2 Bland-Altman plots 17

References **21**

1 Configuration and packages

All the analyses described in this document were performed with R 4.5.1, and the following R packages:

```r
library(cowplot)
library(here)
library(knitr)
library(mice)
library(randomForest)
library(rlist)
library(tidyverse)
```

We also load custom R functions written for this project:

```r
source(here("R", "bland_altman_plot.R"))
source(here("R", "convert_stages_to_numeric.R"))
source(here("R", "train_new_rf.R"))
```

More information about the packages versions can be found below:

```r
print(sessionInfo(), locale = FALSE)
```

```
R version 4.4.1 (2024-06-14)
Platform: aarch64-apple-darwin20
Running under: macOS 15.0.1

Matrix products: default
BLAS:   /Library/Frameworks/R.framework/Versions/4.4-arm64/Resources/lib/libRblas.0.dylib
LAPACK: /Library/Frameworks/R.framework/Versions/4.4-arm64/Resources/lib/libRlapack.dylib;  L

attached base packages:
[1] stats     graphics  grDevices utils     datasets  methods   base

other attached packages:
 [1] lubridate_1.9.3      forcats_1.0.0        stringr_1.5.1
 [4] dplyr_1.1.4          purrr_1.0.2          readr_2.1.5
 [7] tidyr_1.3.1          tibble_3.2.1         ggplot2_3.5.2
[10] tidyverse_2.0.0      rlist_0.4.6.2        randomForest_4.7-1.2
[13] mice_3.18.0          knitr_1.50           here_1.0.1
[16] cowplot_1.2.0

loaded via a namespace (and not attached):
 [1] gtable_0.3.6      shape_1.4.6.1     xfun_0.52         lattice_0.22-6
 [5] tzdb_0.4.0        vctrs_0.6.5       tools_4.4.1       generics_0.1.3
```

```
 [9] fansi_1.0.6        pan_1.9            pkgconfig_2.0.3   jomo_2.7-6
[13] Matrix_1.7-0       data.table_1.16.0 lifecycle_1.0.4   compiler_4.4.1
[17] munsell_0.5.1      codetools_0.2-20   htmltools_0.5.8.1 yaml_2.3.10
[21] glmnet_4.1-8       pillar_1.9.0       nloptr_2.1.1      MASS_7.3-61
[25] iterators_1.0.14   rpart_4.1.23       boot_1.3-31       foreach_1.5.2
[29] mitml_0.4-5        nlme_3.1-164       tidyselect_1.2.1  digest_0.6.37
[33] stringi_1.8.4      splines_4.4.1      rprojroot_2.0.4   fastmap_1.2.0
[37] grid_4.4.1         colorspace_2.1-1   cli_3.6.3         magrittr_2.0.3
[41] survival_3.6-4     utf8_1.2.4         broom_1.0.7       withr_3.0.1
[45] scales_1.3.0       backports_1.5.0    timechange_0.3.0  rmarkdown_2.28
[49] nnet_7.3-19        lme4_1.1-35.5      hms_1.1.3         evaluate_1.0.0
[53] rlang_1.1.4        Rcpp_1.0.13        glue_1.8.0        rstudioapi_0.17.1
[57] minqa_1.2.8        jsonlite_1.8.9     R6_2.5.1
```

2 Load and refactor data

2.1 Individual data

We first load the data contained in a CSV file:

```r
dat <- read.csv(
  file = here("data", "data.csv"),
  row.names = 1,
  stringsAsFactors = TRUE,
  na.strings = c("", "NA")
) |>
  mutate(across(I1:M2, as.ordered))
```

We display a summary of the dataset at this stage:

```r
summary(dat, maxsum = 9)
```

```
     sex            age             agenesis      nb_agenesis        I1
 Female:360   Min.   : 6.50   35       : 46   Min.    :0.000   F   :  8
 Male  :266   1st Qu.:10.40   45 35    : 44   1st Qu.:0.000   G   : 65
              Median :12.00   12 22    : 38   Median :0.000   H   :547
              Mean   :11.72   45       : 29   Mean    :0.885   NA's:  6
              3rd Qu.:13.00   12       : 20   3rd Qu.:1.000
              Max.   :15.00   22       : 16   Max.    :8.000
                              42       : 14
                              (Other):104
                              NA's    :315
    I2          C          PM1         PM2        M1          M2
 E   :  2   E   : 22   E   : 71   B   :  1   F: 10   C   :  1
```

```
 F    : 22   F    :198   F    :196   C    :  1   G:102   D    : 52
 G    : 98   G    :196   G    :161   D    : 31   H:514   E    :147
 H    :499   H    :209   H    :197   E    :108           F    :230
 NA's:  5    NA's:  1    NA's:  1    F    :218           G    :179
                                     G    :124           H    : 13
                                     H    : 79           NA's:  4
                                     NA's: 64
```

2.2 Tables of conversion of dental stages by sex

At this point, we import the sex-specific conversion tables published by Demirjian, Goldstein, and Tanner (1973). We retained the 1973 standard because of this decile-level resolution; the 1976 revision contains only a graphical percentile chart and omits the underlying numeric values.

For each sex, the first table assigns a numerical maturity score to each radiographic stage (A–H) of the seven left-mandibular permanent teeth. Let's begin with boys:

```
## Tables for boys:
stages_boys <- read.csv2(
  file = here("data", "stages_boys.csv"),
  row.names = 1
)
print(stages_boys)
```

```
    M2   M1  PM2  PM1    C   I2   I1
A  2.1  0.0  1.7  0.0  0.0  0.0  0.0
B  3.5  0.0  3.1  0.0  0.0  0.0  0.0
C  5.9  0.0  5.4  3.4  0.0  0.0  0.0
D 10.1  8.0  9.7  7.0  3.5  3.2  0.0
E 12.5  9.6 12.0 11.0  7.9  5.2  1.9
F 13.2 12.3 12.8 12.3 10.0  7.8  4.1
G 13.6 17.0 13.2 12.7 11.0 11.7  8.2
H 15.4 19.3 14.4 13.5 11.9 13.7 11.8
```

Then, the second table converts the global maturity score (obtained by summing the seven tooth scores for an individual) into dental age, expressed in years.

```
score_to_age_boys <- read.csv2(
  file = here("data", "score_to_age_boys.csv")
)
head(score_to_age_boys)
```

```
  Score Age
1  12.4 3.0
2  12.9 3.1
3  13.5 3.2
4  14.0 3.3
5  14.5 3.4
6  15.0 3.5
```

We also load the same data for girls:

```r
## Table for girls:
stages_girls <- read.csv2(
  file = here("data", "stages_girls.csv"),
  row.names = 1
)
print(stages_girls)
```

```
    M2   M1  PM2  PM1    C   I2   I1
A  2.7  0.0  1.8  0.0  0.0  0.0  0.0
B  3.9  0.0  3.4  0.0  0.0  0.0  0.0
C  6.9  0.0  6.5  3.7  0.0  0.0  0.0
D 11.1  4.5 10.6  7.5  3.8  3.2  0.0
E 13.5  6.2 12.7 11.8  7.3  5.6  2.4
F 14.2  9.0 13.5 13.1 10.3  8.0  5.1
G 14.5 14.0 13.8 13.4 11.6 12.2  9.3
H 15.6 16.2 14.6 14.1 12.4 14.2 12.9
```

```r
score_to_age_girls <- read.csv2(
  file = here("data", "score_to_age_girls.csv")
)
head(score_to_age_girls)
```

```
  Score Age
1  13.7 3.0
2  14.4 3.1
3  15.1 3.2
4  15.8 3.3
5  16.6 3.4
6  17.3 3.5
```

3 Compute the dental age in the dataset

3.1 Convert stages into numeric values

Here, we convert each radiographic stage (A–H) in the dataset to its sex- and tooth-specific numerical score, using the first Demirjian table loaded earlier, i.e. stages_boys or stages_girls, as appropriate.

```
dat.num <- matrix(NA, nrow = nrow(dat), ncol = 7) |>
  as.data.frame()
colnames(dat.num) <- colnames(select(dat, I1:M2))
rownames(dat.num) <- rownames(dat)
for (i in 1:nrow(dat.num)) {
  for (j in colnames(dat.num)) {
    dat.num[i, j] <- convert_stages_to_numeric(
      stage = as.character(dat[i, j]),
      tooth = j,
      sex = as.character(dat[i, "sex"]),
      stages_boys = stages_boys,
      stages_girls = stages_girls
    )
  }
}
colnames(dat.num) <- paste0(colnames(dat.num), "_numeric")
dat <- data.frame(dat, dat.num)
head(dat)
```

```
        sex  age agenesis nb_agenesis I1 I2 C PM1  PM2 M1 M2 I1_numeric
4986 Female  9.2       15           1  H  G F   F    E  H  D       12.9
5323   Male 14.5       35           1  H  H H   H    F  H  G       11.8
5423   Male  9.4       35           1  G  G F   E    E  H  E        8.2
5480 Female  7.5    15 25           2  F  F F   E    E  G  E        5.1
5634 Female 13.7       45           1  H  H G   H    F  H  G       12.9
5799 Female 14.5    45 35           2  H  H G   H <NA>  H  G       12.9
     I2_numeric C_numeric PM1_numeric PM2_numeric M1_numeric M2_numeric
4986       12.2      10.3        13.1        12.7       16.2       11.1
5323       13.7      11.9        13.5        12.8       19.3       13.6
5423       11.7      10.0        11.0        12.0       19.3       12.5
5480        8.0      10.3        11.8        12.7       14.0       13.5
5634       14.2      11.6        14.1        13.5       16.2       14.5
5799       14.2      11.6        14.1          NA       16.2       14.5
```

Once every stage has been converted to its numeric score, we compute the sum of these values row-wise, to give a global maturity score for each individual:

```r
dat$Sum <- dat |>
  select(I1_numeric:M2_numeric) |>
  rowSums()
head(dat)
```

```
        sex  age agenesis nb_agenesis I1 I2 C PM1  PM2 M1 M2 I1_numeric
4986 Female  9.2       15           1  H  G F   F    E  H  D       12.9
5323   Male 14.5       35           1  H  H H   H    F  H  G       11.8
5423   Male  9.4       35           1  G  G F   E    E  H  E        8.2
5480 Female  7.5    15 25           2  F  F F   E    E  G  E        5.1
5634 Female 13.7       45           1  H  H G   H    F  H  G       12.9
5799 Female 14.5    45 35           2  H  H G   H <NA>  H  G       12.9
     I2_numeric C_numeric PM1_numeric PM2_numeric M1_numeric M2_numeric  Sum
4986       12.2      10.3        13.1        12.7       16.2       11.1 88.5
5323       13.7      11.9        13.5        12.8       19.3       13.6 96.6
5423       11.7      10.0        11.0        12.0       19.3       12.5 84.7
5480        8.0      10.3        11.8        12.7       14.0       13.5 75.4
5634       14.2      11.6        14.1        13.5       16.2       14.5 97.0
5799       14.2      11.6        14.1          NA       16.2       14.5   NA
```

3.2 Computation of dental age

Finally, we compute a dental age from the `Sum` variable obtained above using the second sex-specific Demirjian table (score_to_age_boys or score_to_age_girls). Because the Demirjian tables provide ages only at 0.1-year intervals, any summed score that falls between two tabulated values is assigned a continuous dental age obtained by linear interpolation between the two closest age points. No extrapolation beyond the published limits is applied.

```r
dat <- mutate(
  dat,
  DA = ifelse(
    sex == "Male" & between(Sum, min(score_to_age_boys$Score),
                                 max(score_to_age_boys$Score)),
    yes = approx(score_to_age_boys$Score, score_to_age_boys$Age,
                 Sum, rule = 2)$y,
    no = ifelse(
      sex == "Female" & between(Sum, min(score_to_age_girls$Score),
                                     max(score_to_age_girls$Score)),
      yes = approx(score_to_age_girls$Score, score_to_age_girls$Age,
                   Sum, rule = 2)$y,
      no = NA
    )
  )
)
head(dat)
```

```
          sex  age agenesis nb_agenesis I1 I2 C PM1  PM2 M1 M2 I1_numeric
4986 Female  9.2       15           1  H  G F   F    E  H  D       12.9
5323   Male 14.5       35           1  H  H H   H    F  H  G       11.8
5423   Male  9.4       35           1  G  G F   E    E  H  E        8.2
5480 Female  7.5    15 25           2  F  F F   E    E  G  E        5.1
5634 Female 13.7       45           1  H  H G   H    F  H  G       12.9
5799 Female 14.5    45 35           2  H  H G   H <NA>  H  G       12.9
     I2_numeric C_numeric PM1_numeric PM2_numeric M1_numeric M2_numeric  Sum
4986       12.2      10.3        13.1        12.7       16.2       11.1 88.5
5323       13.7      11.9        13.5        12.8       19.3       13.6 96.6
5423       11.7      10.0        11.0        12.0       19.3       12.5 84.7
5480        8.0      10.3        11.8        12.7       14.0       13.5 75.4
5634       14.2      11.6        14.1        13.5       16.2       14.5 97.0
5799       14.2      11.6        14.1          NA       16.2       14.5   NA
            DA
4986  9.240000
5323 14.000000
5423  9.157143
5480  7.820000
5634 12.700000
5799        NA
```

Finally, we count for each individual the number of missing stage ratings because of bilateral agenesis:

```
dat <- dat %>%
  mutate(NbNA = rowSums(is.na(select(., I1:M2))))
```

4 Split the data according to sex

We remove all instrumental columns created above, which won't be used in subsequent analyses. At the same time, we create below two subsets of the dataset, to allow separate inspection of missing data patterns for boys and girls respectively.

```
dat <- select(dat, -ends_with("numeric"))
datF <- subset(dat, sex == "Female")
datM <- subset(dat, sex == "Male")
```

5 Inspecting missing data patterns

```
pattern.tot <- dat |>
  select(I1:M2) |>
  md.pattern()
```

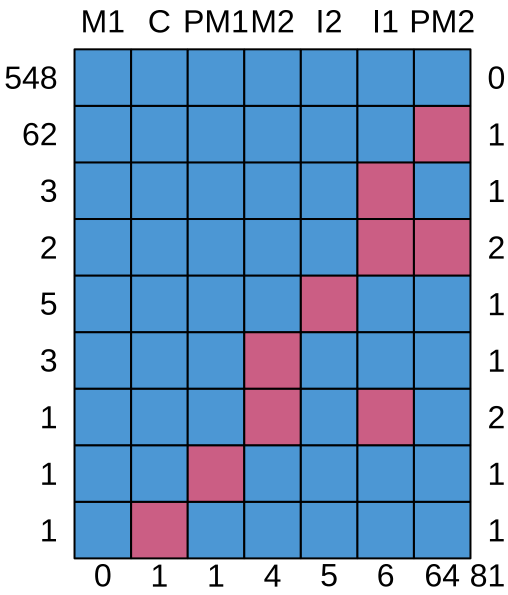

Figure S1: Missing data patterns for the whole dataset.

```
pattern.boys <- datM |>
  select(I1:M2) |>
  md.pattern()
```

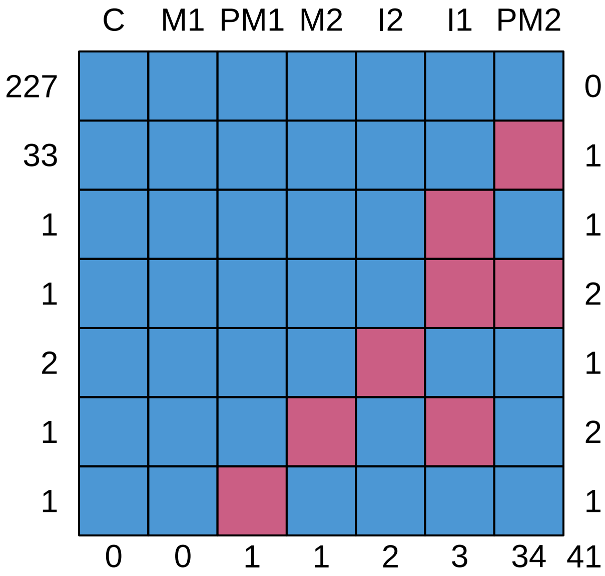

Figure S2: Missing data patterns for boys only.

```
pattern.girls <- datF |>
  select(I1:M2) |>
  md.pattern()
```

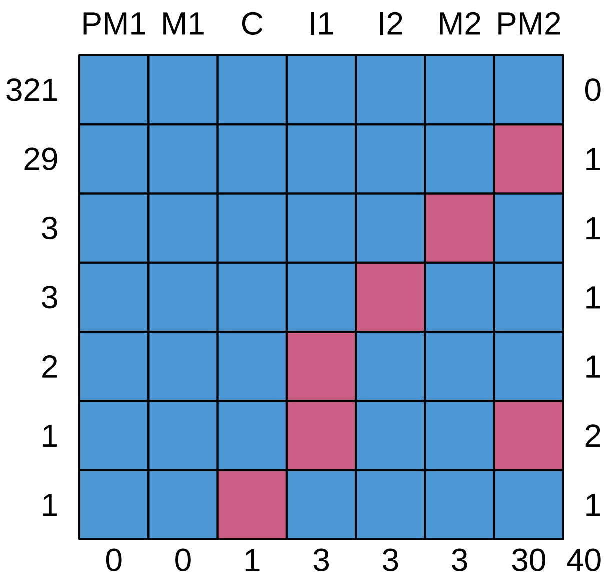

Figure S3: Missing data patterns for girls only.

6 Dental age estimation using random forests

6.1 Pipeline design

At this stage, we have 548 complete individuals whose dental age could be estimated using Demirjian's method, and 78 individuals with at least one missing value (i.e., at least one bilateral agenesis) whose dental age is still to be estimated. In this section, we use random forests to estimate the dental age of these 78 individuals (Breiman (2001)). For each one of these individuals, we proceed as follows:

1. Inspect the pattern of missingness on this target individual.
2. Using the reference sample of 548 complete individuals, train a random forest model *using only the teeth that are non-missing on the target individual.*
3. Check and assess the performance of this random forest model.
4. Apply this random forest model on the target individual, thus predicting a dental age.

Furthermore, we split upstream the reference sample into two subsets: a training sample composed of 80% of the individuals (taken at random, used for step 2), and a testing sample composed of the remaining 20% of individuals (used for step 3).

We create all subsets described above with the following code chunk:

```
## Reference sample of complete individuals:
ref <- dat |>
  subset(NbNA == 0) |>
  select(DA, sex, I1:M2)

## Training sample to build RF models:
set.seed(2025)
train.indivs <- sample(
  x = rownames(ref),
  size = 438,         # 80% of the total sample
  replace = FALSE
)
training <- ref[train.indivs, ]

## testing sample:
testing <- ref[! rownames(ref) %in% train.indivs, ]

## Sample of individuals whose dental age is to be estimated:
targets <- subset(dat, NbNA > 0) |>
  subset(NbNA > 0) |>
  select(DA, sex, I1:M2)
```

6.2 Train random forests models

Since there are only eight patterns of missingness in the data (see Figure S1), only eight different random forest models are needed to estimate the dental age of all 78 target individuals. In the loop below, we consider sequentially each target individual: if their pattern of missingness had not been found earlier in the loop, then a new random forest model is built for this precise set of teeth. Otherwise, if their pattern of missingness has already been observed earlier, we simply re-use the corresponding random forest model, thus saving computation time.

For every model, predictive performance is internally validated with the out-of-bag (OOB) error provided by the random-forest algorithm , i.e. the mean error computed on the bootstrap-excluded observations at each tree.

All random forest models are stored in the object `list.model` for further inspection later on.

```
## Initialize a list of RF models:
list.models <- list()

## Dental age estimation:
for (i in 1:nrow(targets)) { # for each target individual:
  ## Record missingness pattern:
  target <- targets[i, -1, drop = FALSE]
```

```r
  miss.patt <- paste(colnames(target)[is.na(target)],
                     collapse = "_")

  ## Keep only the non-missing teeth:
  target <- target[1, !is.na(target), drop = FALSE]
  iref <- training[, c("DA", colnames(target))]

  ## If this pattern is not known yet, train a new RF model:
  if (! miss.patt %in% names(list.models)) {
    rf.mod <- train_new_rf(
      learning = iref,
      testing = testing,
      target = target,
      include.sex = TRUE
    )
    list.models <- rlist::list.append(
      list.models,
      rf.mod
    )
    names(list.models)[length(list.models)] <- miss.patt
    targets[i, "DA"] <- predict(rf.mod$rf, target)
  } else { # if the missingness pattern is known, apply the corresp. model:
    rf.mod <- list.models[[miss.patt]]
    targets[i, "DA"] <- predict(rf.mod$rf, target)
  }
}

## Save all results in R objects:
saveRDS(
  object = list.models,
  file = here("objects", "list.models.rds")
)
saveRDS(
  object = targets,
  file = here("objects", "targets.rds")
)
```

6.3 Variable importance in random forests models

In Figure S4, we can see that for all random forest models, the sex has only a moderate impact (although not totally negligible in some cases) on dental age estimation.

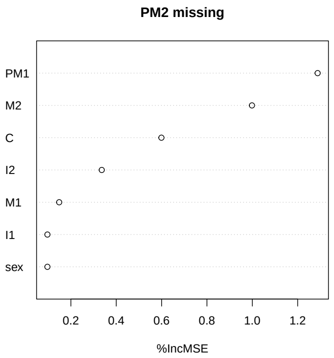

(a) PM2 missing

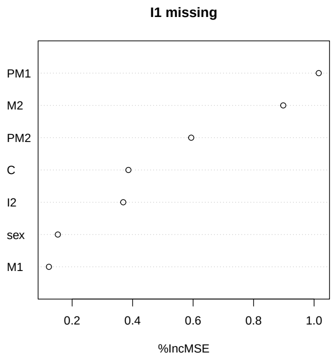

(b) I1 missing

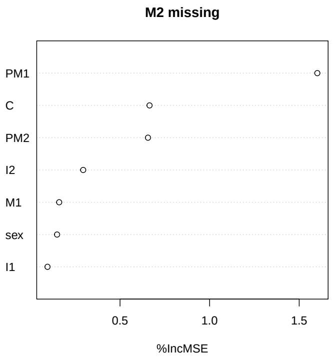

(c) M2 missing

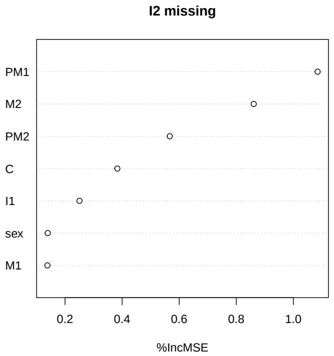

(d) I2 missing

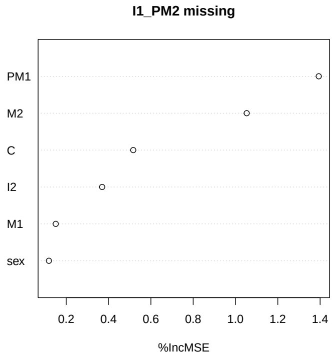

(e) I1 and PM2 missing

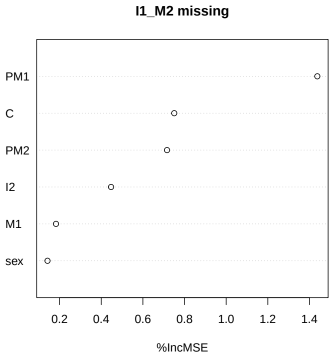

(f) I1 and M2 missing

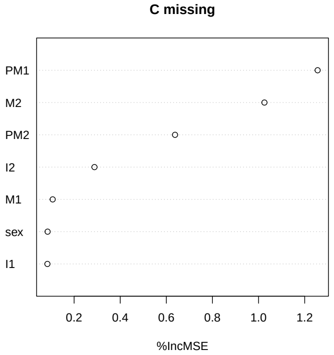

(g) C missing

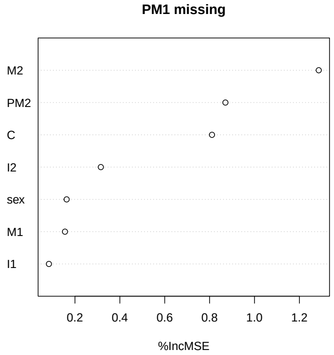

(h) PM1 missing

Figure S4: Variable importance for all eight random forest models, computed as the increase in mean square error when the corresponding variable is submitted to random permutations before building the model.

```r
for (k in 1:length(list.models)) {
  varImpPlot(
    list.models[[k]]$rf,
    main = paste(names(list.models)[k], "missing"),
    type = 1,
    scale = FALSE
  )
}
```

6.4 Results of dental age estimation

The full results can be found in Table S1.

```r
kable(targets)
```

Table S1: Results of dental age estimation by random forests for each individual with at least one missing value. Estimated dental age is given in the column DA.

	DA	sex	I1	I2	C	PM1	PM2	M1	M2
5799	13.315321	Female	H	H	G	H	NA	H	G
6123	10.984272	Male	H	H	F	F	NA	H	E
6129	13.629019	Female	H	H	H	H	NA	H	F
6340	10.685615	Female	H	H	F	F	NA	H	E
6374	12.386617	Male	H	H	G	H	NA	H	E
6869	7.927832	Female	G	F	E	E	NA	G	D
6918	12.118661	Female	H	H	H	F	NA	H	F
7356	8.798657	Male	G	G	F	E	NA	H	D
7463	12.796474	Male	H	H	H	H	NA	H	E
7506	10.563403	Male	H	G	F	F	NA	H	F
7507	10.563403	Male	H	G	F	F	NA	H	F
7548	8.933255	Male	G	H	F	E	NA	G	D
7554	11.443098	Female	H	H	G	G	NA	H	E
7588	11.638587	Female	H	H	G	F	NA	H	F
7845	8.105390	Female	NA	G	F	E	D	G	D
7943	11.734434	Female	H	H	G	G	F	H	NA
8049	12.539894	Female	H	H	H	G	NA	H	F
8134	13.109851	Female	H	H	H	G	NA	H	G
8289	13.629019	Female	H	H	H	H	NA	H	F
8759	15.192311	Male	H	H	H	H	NA	H	H
9019	10.604244	Female	NA	H	G	F	E	H	E
9024	10.459300	Female	H	H	F	E	NA	H	E
9028	12.270326	Male	H	H	G	G	NA	H	F
9040	10.984272	Male	H	H	F	F	NA	H	E
9149	10.393075	Male	H	NA	F	E	E	H	E
9309	11.370497	Male	H	H	F	F	NA	H	F
9354	11.333580	Male	H	H	G	F	NA	H	E

Table S1: Results of dental age estimation by random forests for each individual with at least one missing value. Estimated dental age is given in the column DA.

	DA	sex	I1	I2	C	PM1	PM2	M1	M2
9829	10.664529	Female	NA	H	F	F	NA	H	E
9937	9.068005	Male	H	G	E	E	NA	G	E
9970	11.638587	Female	H	H	G	F	NA	H	F
10128	12.660168	Male	H	H	G	G	NA	H	G
10212	10.550961	Male	NA	G	G	F	NA	H	F
10385	14.184294	Female	H	H	H	H	NA	H	G
10388	9.581154	Male	H	G	F	F	NA	G	E
10532	11.064377	Female	H	H	F	F	NA	H	F
10554	10.393660	Male	NA	H	F	F	E	H	D
10598	10.984272	Male	H	H	F	F	NA	H	E
10939	11.609802	Female	H	NA	H	F	E	H	F
11231	10.962639	Male	NA	G	G	G	F	H	NA
11235	14.184294	Female	H	H	H	H	NA	H	G
11398	10.984272	Male	H	H	F	F	NA	H	E
11477	13.428729	Male	H	H	H	G	NA	H	G
11504	10.984272	Male	H	H	F	F	NA	H	E
11656	10.685615	Female	H	H	F	F	NA	H	E
11852	11.760972	Male	H	H	F	G	NA	H	F
11902	12.211389	Female	H	H	G	G	NA	H	G
12032	13.428729	Male	H	H	H	G	NA	H	G
12148	13.725456	Male	H	H	G	H	NA	H	G
12279	8.142920	Female	G	F	E	E	NA	G	E
12492	10.510411	Male	H	H	F	E	NA	H	E
12687	15.192311	Male	H	H	H	H	NA	H	H
12700	14.501939	Female	H	H	NA	H	H	H	G
12734	10.206274	Female	H	NA	G	F	F	G	E
13003	10.881573	Female	H	H	G	F	NA	H	E
13101	12.371866	Male	H	H	G	NA	F	H	F
13264	12.592810	Female	H	H	H	H	NA	H	E
13566	8.939546	Male	H	G	F	F	NA	G	D
13970	11.876126	Female	H	H	G	G	NA	H	F
14467	9.032204	Female	G	G	F	F	NA	H	E
14623	11.876126	Female	H	H	G	G	NA	H	F
14825	10.990000	Male	H	NA	F	F	F	H	E
14928	8.037861	Female	G	NA	F	E	D	G	D
15020	13.725456	Male	H	H	G	H	NA	H	G
15054	11.760972	Male	H	H	F	G	NA	H	F
15072	12.169194	Female	H	H	G	G	G	H	NA
15545	13.629019	Female	H	H	H	H	NA	H	F
15645	8.297528	Male	G	F	F	E	NA	G	E
15664	14.184294	Female	H	H	H	H	NA	H	G
15742	14.767917	Male	H	H	H	H	NA	H	G

Table S1: Results of dental age estimation by random forests for each individual with at least one missing value. Estimated dental age is given in the column `DA`.

	DA	sex	I1	I2	C	PM1	PM2	M1	M2
15868	12.211389	Female	H	H	G	G	NA	H	G
16001	11.370497	Male	H	H	F	F	NA	H	F
16297	12.660168	Male	H	H	G	G	NA	H	G
16315	14.184294	Female	H	H	H	H	NA	H	G
16464	9.492004	Male	G	G	F	F	NA	G	F
16551	9.215754	Male	H	G	F	E	NA	G	E
16973	14.872985	Female	H	H	H	H	H	H	NA
17094	13.315321	Female	H	H	G	H	NA	H	G
17492	14.184294	Female	H	H	H	H	NA	H	G

7 Model diagnostics

7.1 Metrics

Table S2 summarises the results obtained with the training

```
lapply(list.models, \(x) list.extract(x, "metrics.training")) %>%
  do.call(what = rbind, args = .) %>%
   .[, c("ME","SD_ME", "MSE", "SD_MSE","MAE","SD_MAE", "RMSE","R2")] %>%
  round(4) %>%
  kable()
```

Table S2: Performance metrics of all eight random forest models (based on out-of-bag results on the training sample).

	ME	SD_ME	MSE	SD_MSE	MAE	SD_MAE	RMSE	R2
PM2	-0.0215	0.4457	0.1986	0.4244	0.3136	0.3170	0.4457	0.9520
I1	-0.0263	0.2400	0.0582	0.1998	0.1218	0.2084	0.2412	0.9859
M2	-0.0217	0.3160	0.1001	0.2823	0.2159	0.2316	0.3164	0.9758
I2	-0.0253	0.2590	0.0676	0.2452	0.1392	0.2198	0.2600	0.9837
I1_PM2	-0.0220	0.4622	0.2136	0.4293	0.3377	0.3160	0.4622	0.9484
I1_M2	-0.0174	0.3477	0.1209	0.2987	0.2424	0.2496	0.3478	0.9708
C	-0.0214	0.3645	0.1330	0.2082	0.2801	0.2338	0.3647	0.9679
PM1	-0.0268	0.3146	0.0995	0.2636	0.2266	0.2196	0.3154	0.9760

Furthermore, all metrics are still good as for the testing sample (Table S3), and are consistent with those obtained for the training sample.

```r
lapply(list.models, \(x) list.extract(x, "metrics.testing")) %>%
  do.call(what = rbind, args = .) %>%
   .[, c("ME", "MSE", "MAE","RMSE","R2")] %>%
  round(4) %>%
  kable()
```

Table S3: Performance metrics of all eight random forest models for the testing sample.

	ME	MSE	MAE	RMSE	R2
PM2	-0.0009	0.1300	0.2807	0.3606	0.9702
I1	-0.0148	0.0240	0.0824	0.1549	0.9945
M2	0.0153	0.1015	0.2162	0.3186	0.9767
I2	-0.0071	0.0334	0.0992	0.1829	0.9923
I1_PM2	0.0116	0.1327	0.2844	0.3643	0.9696
I1_M2	0.0119	0.1119	0.2372	0.3345	0.9744
C	-0.0298	0.0988	0.2348	0.3144	0.9774
PM1	-0.0172	0.0713	0.1952	0.2670	0.9837

7.2 Bland-Altman plots

Bland–Altman plots were used to graphically evaluate the agreement between the reference DA and the predicted values (Bland and Altman (1986)) both in the training sample (Figure S5) and in the testing sample (Figure S6).

```r
ba.list <- lapply(
  X = list.models,
  FUN = \(x) bland_altman_plot(data = training, model = x$rf)
)
cowplot::plot_grid(
  plotlist = ba.list,
  labels = names(list.models)
)
```

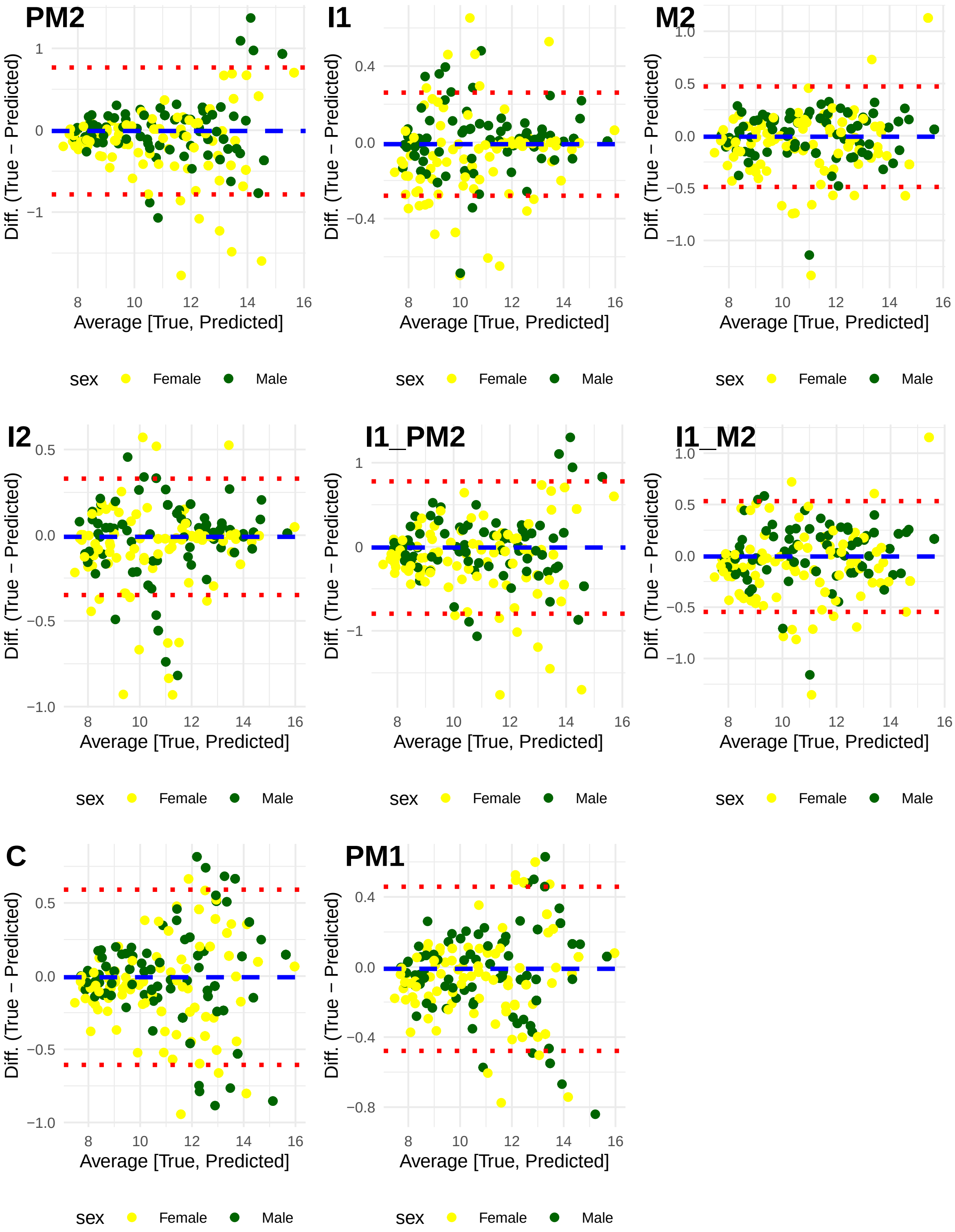

Figure S5: Bland-Altman plots for all eight random forest models in the training sample.

```r
ba.list.test <- lapply(
  X = list.models,
  FUN = \(x) bland_altman_plot(data = testing, model = x$rf)
)
cowplot::plot_grid(
  plotlist = ba.list.test,
  labels = names(list.models)
)
```

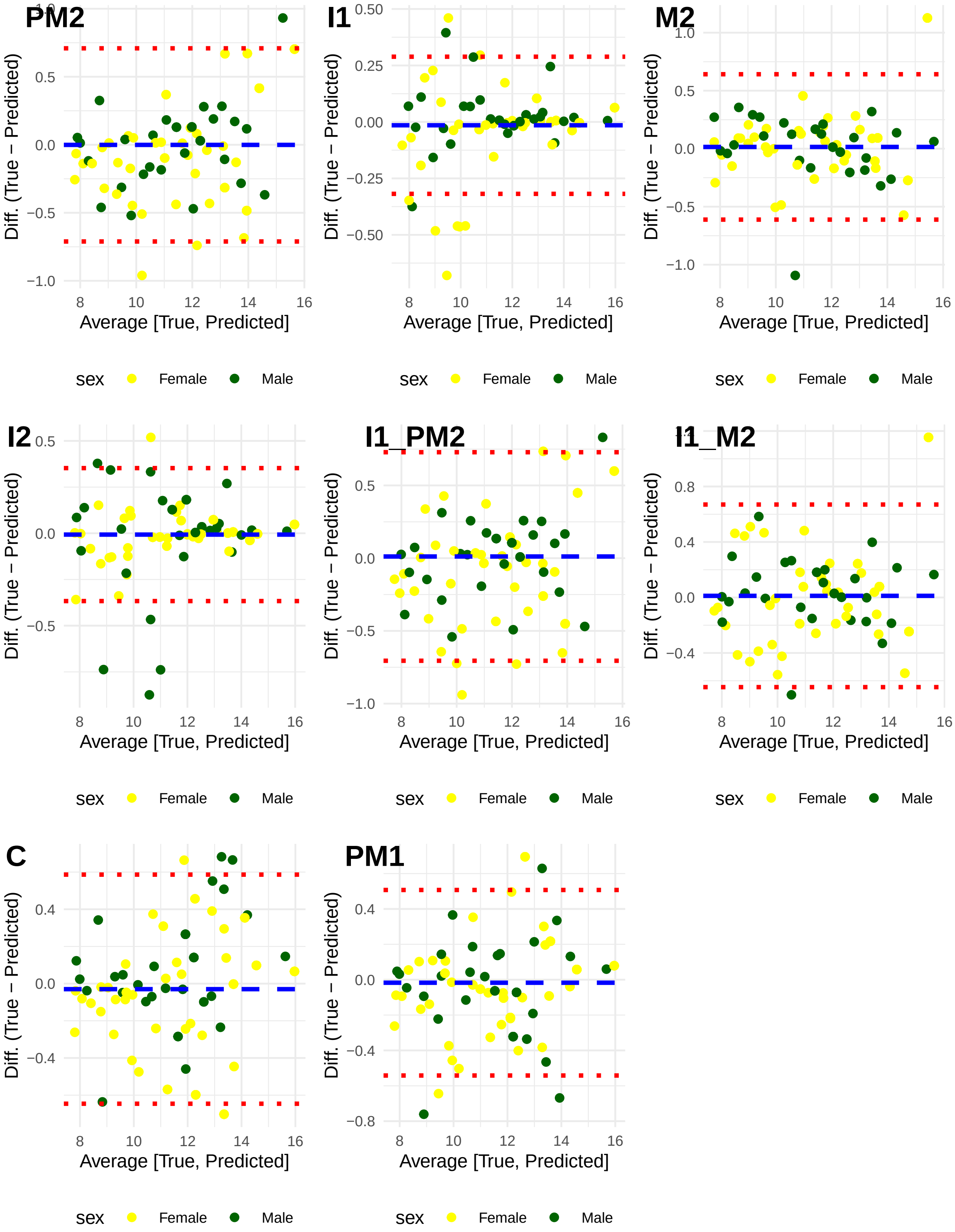

Figure S6: Bland-Altman plots for all eight random forest models in the training sample.

References

Bland, J. M., and D. G. Altman. 1986. "Statistical Methods for Assessing Agreement Between Two Methods of Clinical Measurement." *Lancet (London, England)* 1 (8476): 307–10.

Breiman, Leo. 2001. "Random Forests." *Machine Learning* 45 (1): 5–32. https://doi.org/10.1023/a:1010933404324.

Demirjian, A., H. Goldstein, and J. M. Tanner. 1973. "A New System of Dental Age Assessment." *Human Biology* 45 (2): 211–27.

Table S4. Cohen's kappa coefficients for each mandibular tooth (I1, I2, C, PM1, PM2, M1, M2) and overall inter-rater agreement (mean ± SD).

Tooth	I1	I2	C	PM1	PM2	M1
ϰ	1.000	1.000	0.887	1.000	0.944	1.000

Tooth	M2	Overall (mean ± sd)
ϰ	1.000	0.976 ± 0.044

Table S5. Distribution of the number of congenitally missing teeth (CMT) per affected subject.

Number of CMT	N	Frequency (%)
**1**	160	51.4
**2**	108	34.7
**3**	21	6.8
**4**	11	3.5
**5**	3	1
**6**	3	1
**7**	2	0.6
**8**	3	1

Table S6 : Distribution of tooth agenesis patterns by frequency.

TAC value	Tooth agenesis profile (FDI)	Frequency (%)
**000 000 000 000**	**Control**	50.32
**000 000 016 000**	**35**	7.35
**000 000 016 016**	**35 - 45**	7.19
**002 002 000 000**	**12- 22**	6.07
**000 000 000 016**	**45**	4.63
**002 000 000 000**	**12**	3.19
**000 002 000 000**	**22**	2.56
**000 000 000 002**	**42**	2.24
**016 000 000 000**	**15**	1.76
**016 016 000 000**	**15 - 25**	1.44
**000 016 000 000**	**25**	0.96
**000 000 000 001**	**41**	0.80
**016 016 016 016**	**15 - 25 - 35 - 45**	0.80
**000 000 002 000**	**32**	0.64
**000 000 002 002**	**32 - 42**	0.64
**000 016 016 016**	**25 - 35 - 45**	0.48
**000 000 000 064**	**47**	0.48
**000 000 001 000**	**31**	0.32
**000 016 000 002**	**25 - 42**	0.32
**016 016 016 000**	**15 - 25 - 35**	0.32
**002 002 016 000**	**12 - 22 - 35**	0.32
**004 000 000 000**	**13**	0.32
**000 000 064 000**	**37**	0.32
**000 000 064 064**	**37 - 47**	0.32
**000 000 001 065**	**31 - 41 - 47**	0.16
**016 000 016 000**	**15 - 35**	0.16
**016 000 016 016**	**15 - 35 - 45**	0.16
**000 000 016 002**	**35 - 42**	0.16
**000 016 002 000**	**25 - 32**	0.16
**000 016 016 000**	**25 - 35**	0.16
**016 016 000 016**	**25 - 35 - 45**	0.16
**016 016 008 016**	**15 - 25 - 34 - 45**	0.16
**000 000 016 024**	**35 - 44 - 45**	0.16

**000 000 017 001**	**31 - 35 - 41**	0.16
**000 000 017 018**	**31 - 35 - 42 - 45**	0.16
**000 000 000 018**	**42 - 45**	0.16
**000 000 018 002**	**32 - 35 - 42**	0.16
**000 018 008 000**	**22 - 25 - 34**	0.16
**018 018 016 016**	**12 - 15 - 22 - 25 - 35 - 45**	0.16
**000 024 000 000**	**24 - 25**	0.16
**002 002 001 000**	**12 - 22 - 31**	0.16
**002 002 016 016**	**12 - 22 - 35 - 45**	0.16
**002 004 018 019**	**12 - 23 - 32 - 35 - 41 - 42 - 45**	0.16
**000 002 008 008**	**22 - 34 - 44**	0.16
**000 000 002 018**	**32 - 42 - 45**	0.16
**002 018 000 016**	**12 - 22 - 25 - 45**	0.16
**002 018 021 017**	**12 - 22 - 25- 32- 33 - 35- 41 - 45**	0.16
**024 024 000 016**	**14 - 15 - 24 - 25 - 45**	0.16
**024 024 024 024**	**14 - 15 - 24 - 25 - 34 - 35 - 44 - 45**	0.16
**000 000 004 004**	**33 - 43**	0.16
**004 004 000 016**	**13 - 23 - 45**	0.16
**006 006 000 000**	**12 - 13 - 22 - 23**	0.16
**064 080 000 000**	**17 - 25 - 27**	0.16
**006 006 016 016**	**12 - 13 - 22 - 23 - 35 - 45**	0.16
**000 000 065 065**	**31 - 37 - 41 - 47**	0.16
**000 008 000 016**	**24 - 45**	0.16
**000 000 080 016**	**35 - 37 - 45**	0.16
**008 002 001 001**	**14 - 22 - 31 - 41**	0.16
**080 064 064 064**	**15 - 17 - 27 - 37 - 47**	0.16
**080 080 016 016**	**15 - 17 - 25 - 27 - 35 - 45**	0.16
**008 008 016 000**	**14 - 24 - 35**	0.16
**082 090 000 064**	**12 - 15 - 17 - 22 - 24 - 25 - 27 - 47**	0.16
**090 002 064 000**	**12 - 14 - 15 - 17 - 22 - 37**	0.16
